# Supplementary material for: Detecting known neoepitopes, gene fusions, transposable elements, and circular RNAs in cell-free RNA
Source: Bioinformatics. 2025 May 2;41(5):btaf138. doi: 10.1093/bioinformatics/btaf138 (PMC12057812; doi:10.1093/bioinformatics/btaf138)
Supplement: btaf138_Supplementary_Data [file btaf138_supplementary_data.zip › SupplmentaryFiguresFastNeo.pdf]

Supplmentary Figures:
Detecting known neoepitopes, gene fusions, transposable elements, and circular RNAs in cell-free RNA

Mayank Mahajan and Martin Hemberg

01/23/2025

Figure. S1. (a) A histogram showing the binned frequency of the neoepitope-associated CCDS-nullomers in the double stranded human genome. The number of nullomers in each bin are shown on the Y-axis.(b) A cumulative plot showing the proportion of the neoepitope-associated CCDS-nullomers against the maximum frequency of these CCDS-nullomers in the double stranded human genome.

Figure. S2. Number of putative neoepitopes in the cfRNA of patients grouped by cancer type and healthy using a coverage of (a) 5, (b) 10, (c) 20, and (d) 40 reads per neoepitope. Each dot is coloured by average normalized coverage of all the neoepitopes in the respective cfRNA sample. P-values of two cancer types with most significant differences in distribution of neoepitopes in healthy donors vs. patients are shown. P-values were calculated using the Wilcoxon test with Bonferroni correction. Means and error-bar are shown for each group.

Figure. S3. (a) Number of known neoepitopes detected in plasma cfRNA of pancreatic cancer patients and healthy donors in the GSE136651 dataset. The mean and error-bar are shown for each group. Each dot is colored by average normalized coverage of all the neoepitopes in the respective patient. (b) Heatmap showing the expression of discriminative neoepitopes detected in the cfRNA of pancreatic cancer patients and healthy donors (see Methods). Each column represents a neoepitope, and each row represents a patient or a healthy donor. Column label shows one of the neoepitopes together with the gene symbol. See Supplementary Table S5h for a full list of discriminative neoepitopes with descriptions.

Figure. S4. Heatmap showing the expression of discriminative neoepitopes detected in the cfRNA of 96 healthy donors vs. (a) 54 colorectal cancer, (b) 31 easophagal cancer, (c) 35 lung cancer, (d) 37 stomach cancer, and (e) 10 multiple myeloma patients (see methods). Each column is associated to a neoepitope, and each row is associated to a patient or a healthy donor. Each column represents a neoepitope, and each row represents a patient or a healthy donor. Column label shows one of the neoepitopes together with the gene symbol. See Supplementary Table S5a-f for a full list of discriminative neoepitopes with descriptions.

Figure. S5. (a) Heatmap showing the expression of discriminative neoepitopes detected in the cfRNA of 96 healthy donors vs. 237 cancer patients. Each column is associated to a neoepitope, and each row is associated to a patient or a healthy donor. Each column represents a neoepitope, and each row represents a patient or a healthy donor. Column label shows one of the neoepitopes together with the gene symbol. See Supplementary Table S5g for a full list (b) An Upset plot showing the neoepitopes from the above heatmap that overlap with the discriminative neoepitopes detected in the individual cancer type shown in Table S5a-f.

Figure. S6. An upset plot showing the number of neoepitope-donor pairs discovered using using Lofreq, HaplotypeCaller (HapCall), and bcftools and FastNeo on (a) the GSE142987 dataset and (b) the GSE136651 dataset, and using FastNeo with STAR instead of Bowtie2 in (c) the GSE142987 dataset, and (d) the GSE136651 dataset.

Figure. S7. A line plot showing the runtimes used by Lofreq, HaplotypeCaller (HapCall), and bcftools and FastNeo to analyze the samples in GSE142987 and GSE136651 dataset.

Figure. S8. Number of putative fusions in the cfRNA of patients grouped by cancer type and healthy using a coverage of (a) 3, (b) 5, and (c) 10 reads per neoepitope. Each dot is coloured by average normalized coverage of all the neoepitopes in the respective cfRNA sample. Patients with NAB2-STAT6 gene fusions are coloured as per legend.

Figure. S9. A box and whisker plot showing the TEs that are differentially expressed (p-value <1e-07) in the cfRNA of healthy donors vs. pancreatic cancer patients are shown. Only 4 of the 15 differentially expressed Alu TEs are shown. The outliers with RKPM score ≤1e-10 were assumed to have RKPM score =1e-10. The horizontal bar corresponds to the median, two hinges correspond to the 25th and 75th percentiles, and two whiskers correspond to the largest and smallest value no further than 1.5 times the distance between 25th and 75th percentiles.

Figure. S10. (a) Number of predicted circRNAs in the cfRNA of patients grouped by cancer type and healthy. Each dot is coloured by cumulative CPM of all the circRNAs in the respective cfRNA sample. P-values of two cancer types with most significant differences in distribution of circRNAs in healthy donors vs. patients are shown. P-values were calculated using the Wilcoxon test with Bonferroni correction. Means and error-bar are shown for each group. (b) First two components of a PCA using log of CPM scores of all circRNAs detected in the colorectal cancer patients and healthy donors.

Figure. S11. Overlapped ROCs from all 10 iterations of each of the 5 folds while classifying (a,c,e,g) HCC patients and (b,d,f,h) colorectal cancer patients among the healthy donors. The classifier was trained using random forest on the expression of (a-b) neoepitopes, (c-d) TEs, and (e-f) circRNAs, and (g-h) neoepitopes + TEs in the cfRNA samples. Each ROC line was plotted with alpha=0.1 and the specificity of 0.8 is marked with a solid vertical line. Sensitivity modeled by fitting a generalized additive model to all the ROCs is shown as a dotted green curve, and the sensitivity predicted at a specificity of 0.8 is shown as a green label on the y-axis.

Figure S1

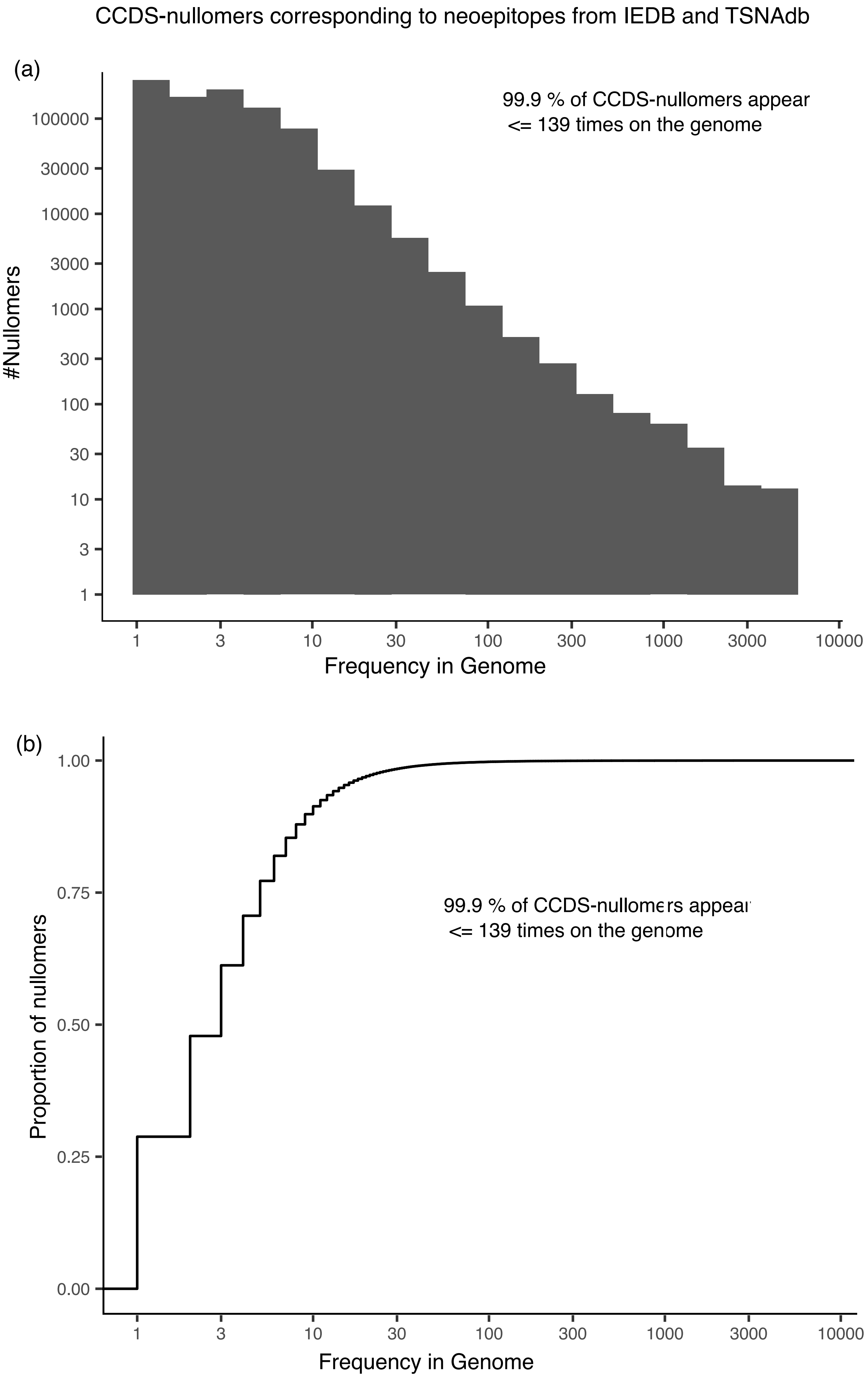
CCDS-nullomers corresponding to neoepitopes from IEDB and TSNAdb
(a)
99.9 % of CCDS-nullomers appear
<= 139 times on the genome
#Nullomers
100000
30000
10000
3000
1000
300
100
30
10
3
1
1
3
10
30
100
300
1000
3000
10000
Frequency in Genome
(b)
99.9 % of CCDS-nullomers appear
<= 139 times on the genome
Proportion of nullomers
1.00
0.75
0.50
0.25
0.00
1
3
10
30
100
300
1000
3000
10000
Frequency in Genome

Figure S2

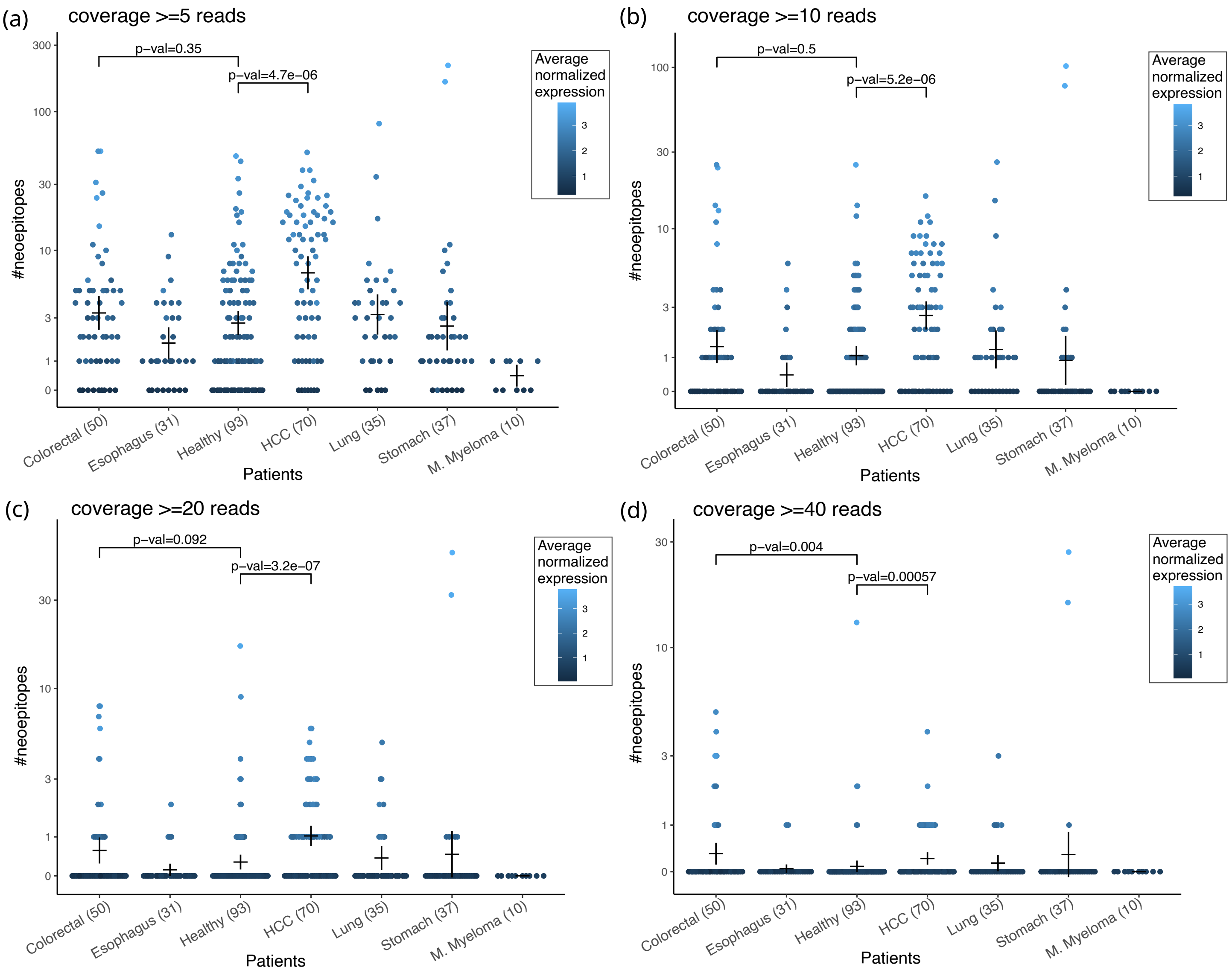
(a)
coverage >=5 reads
p−val=0.35
p−val=4.7e−06
#neoepitopes
Patients
Colorectal (50)
Esophagus (31)
Healthy (93)
HCC (70)
Lung (35)
Stomach (37)
M. Myeloma (10)
Average normalized expression
(b)
coverage >=10 reads
p−val=0.5
p−val=5.2e−06
(c)
coverage >=20 reads
p−val=0.092
p−val=3.2e−07
(d)
coverage >=40 reads
p−val=0.004
p−val=0.00057

Figure S3

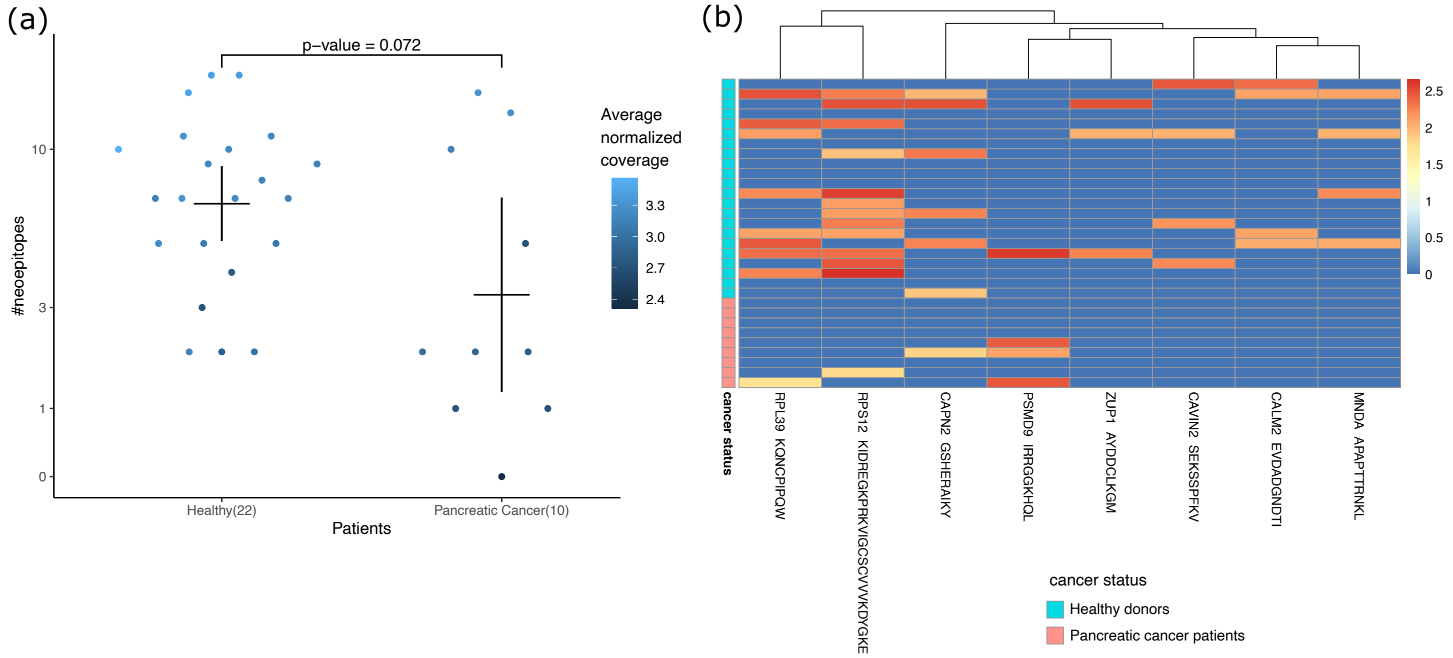
(a)
p–value = 0.072
#neoepitopes
10
3
1
0
Healthy(22)
Pancreatic Cancer(10)
Patients
Average
normalized
coverage
3.3
3.0
2.7
2.4
(b)
2.5
2
1.5
1
0.5
0
cancer status
RPL39_KQNCPIPQW
RPS12_KIDREGKPRKVIGCSCVVVKDYGKE
CAPN2_GSHERAIKY
PSMD9_IRRGGKHQL
ZUP1_AYDDCLKGM
CAVIN2_SEKSSPFKV
CALM2_EVDADGNDTI
MNDA_APAPTTRNKL
cancer status
Healthy donors
Pancreatic cancer patients

Figure S4a-b

(a) Colorectal cancer

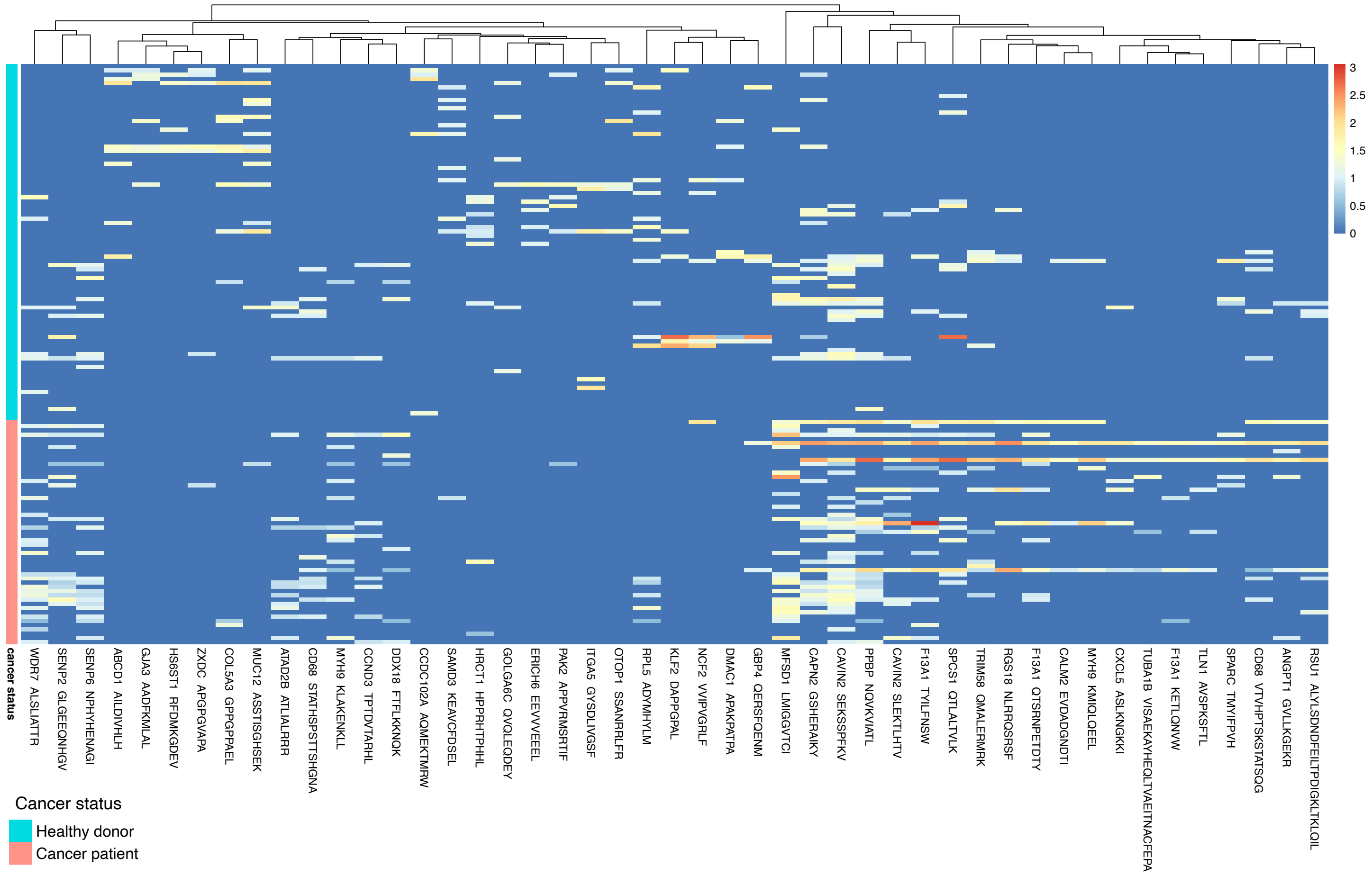

(b) Esophagus cancer

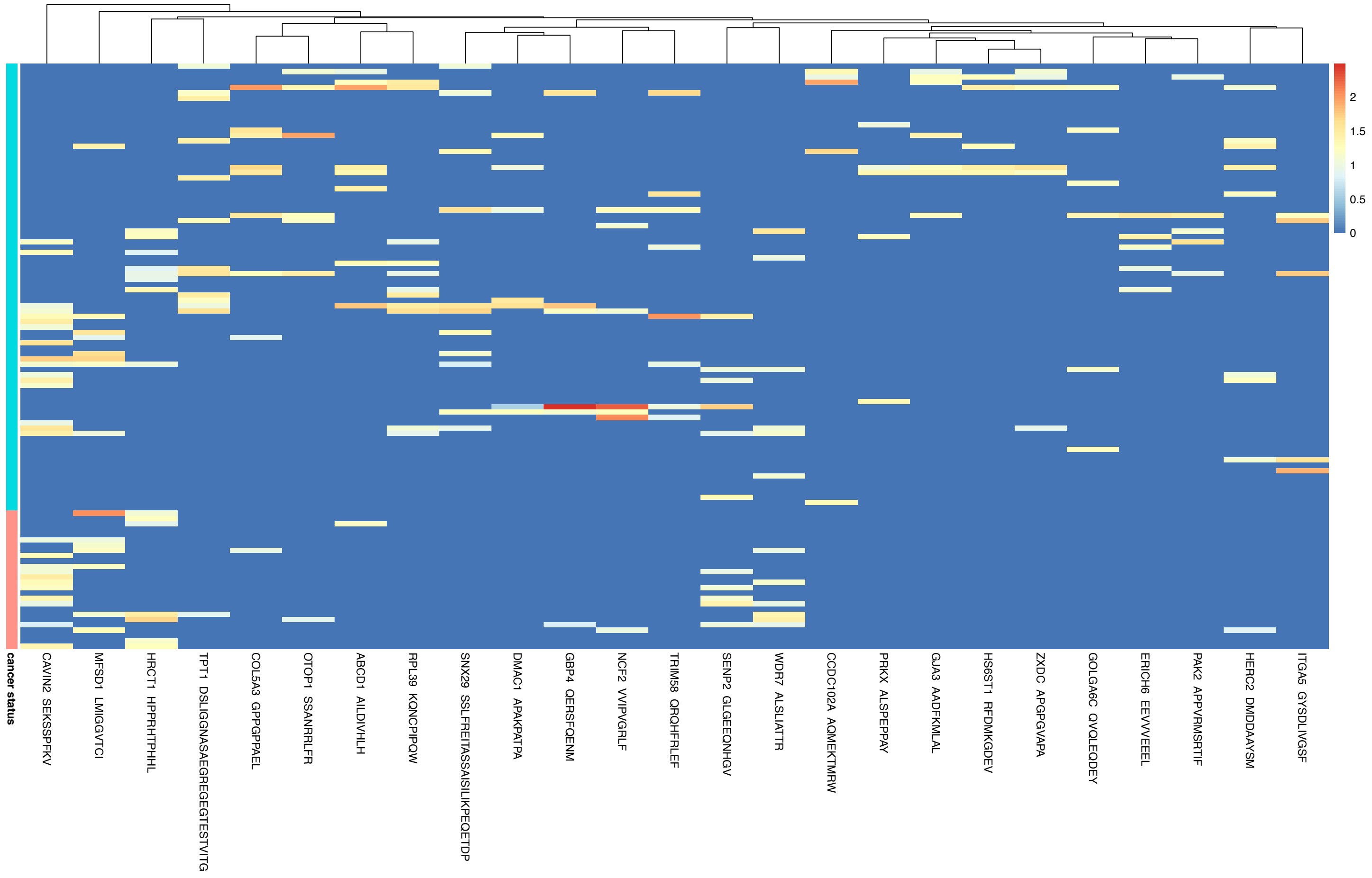

Figure S4c-d

(c) Lung cancer

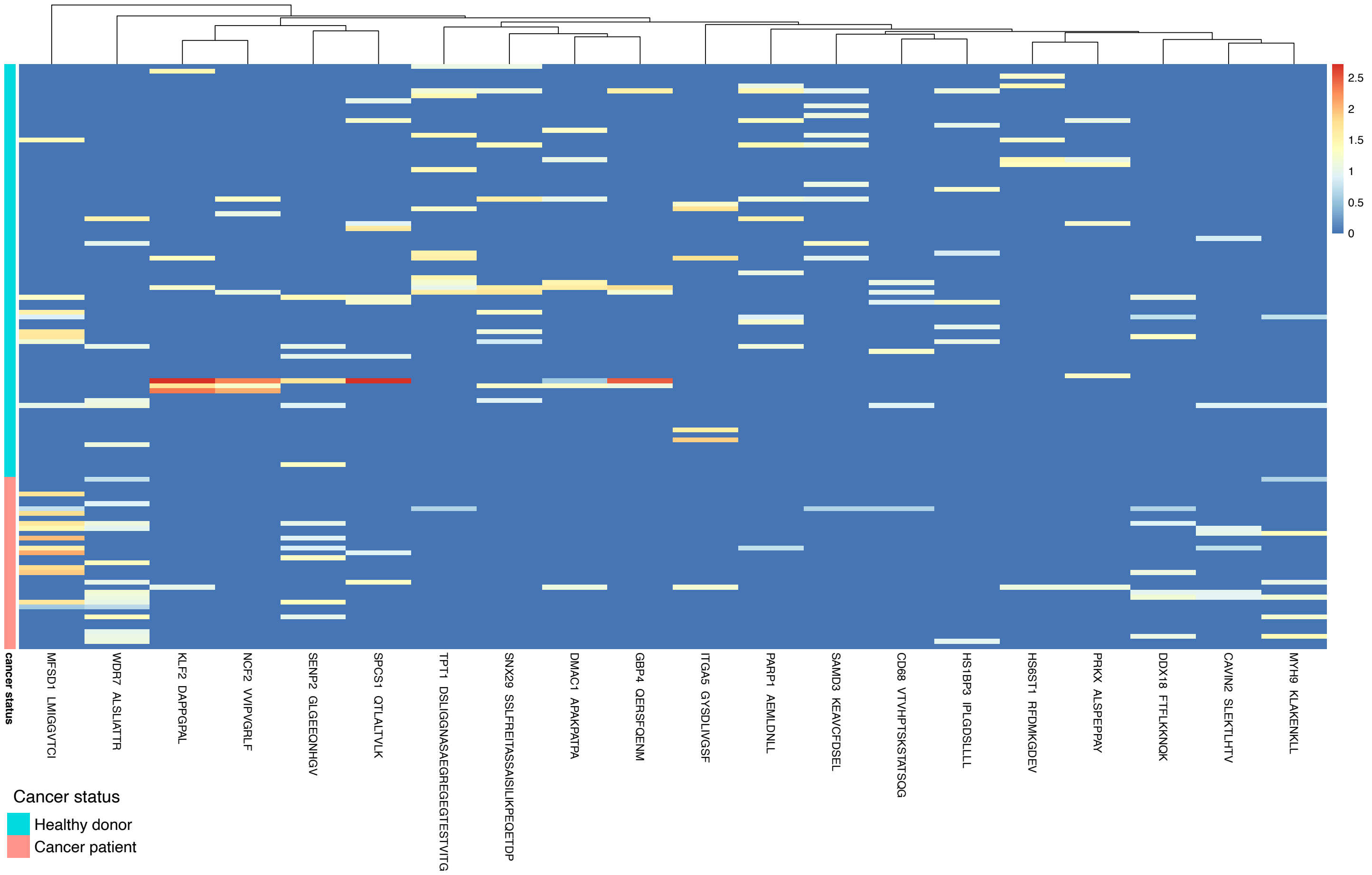

(d) Stomach cancer

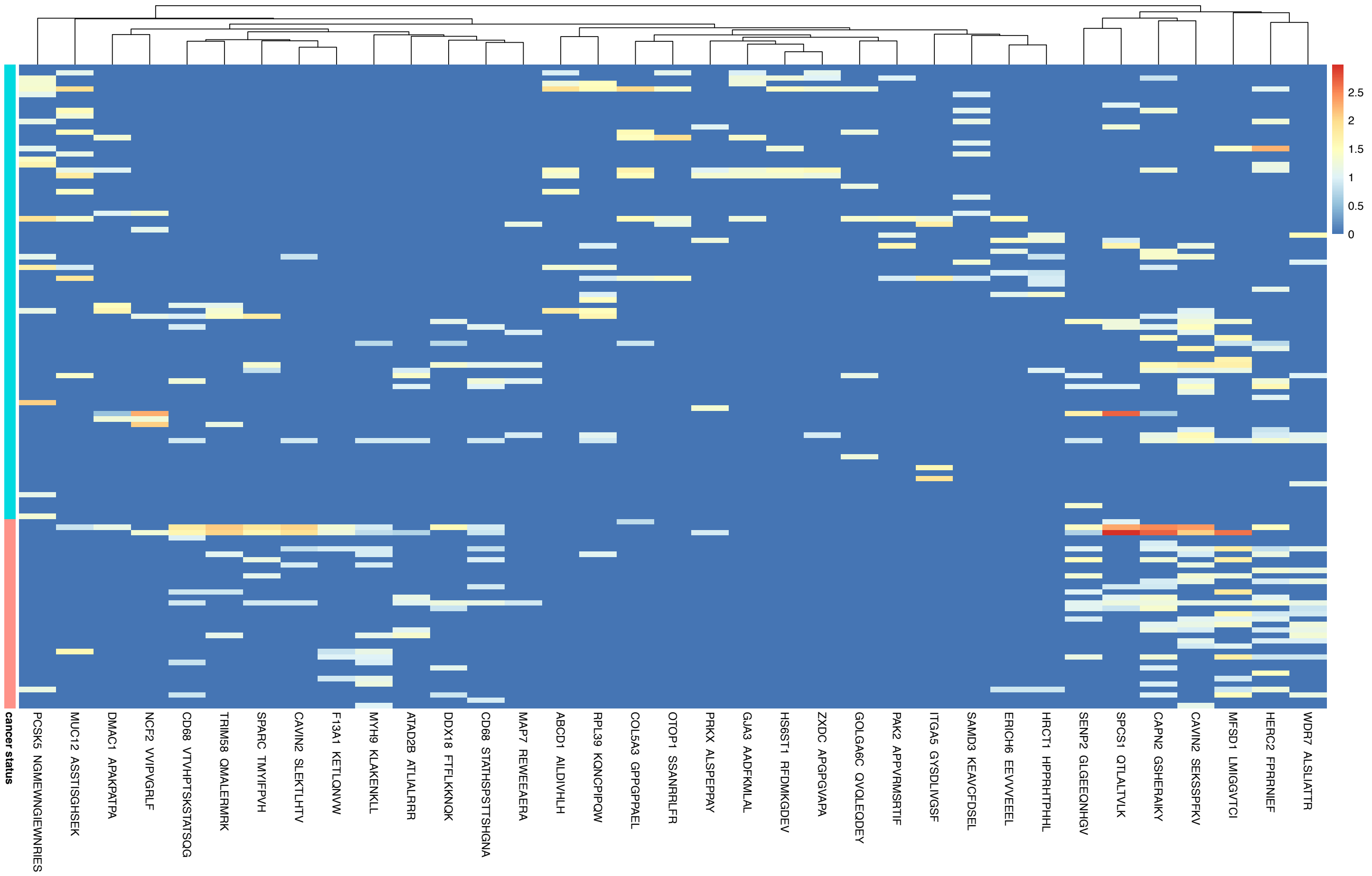

Figure S4e

(e) M.Myeloma cancer

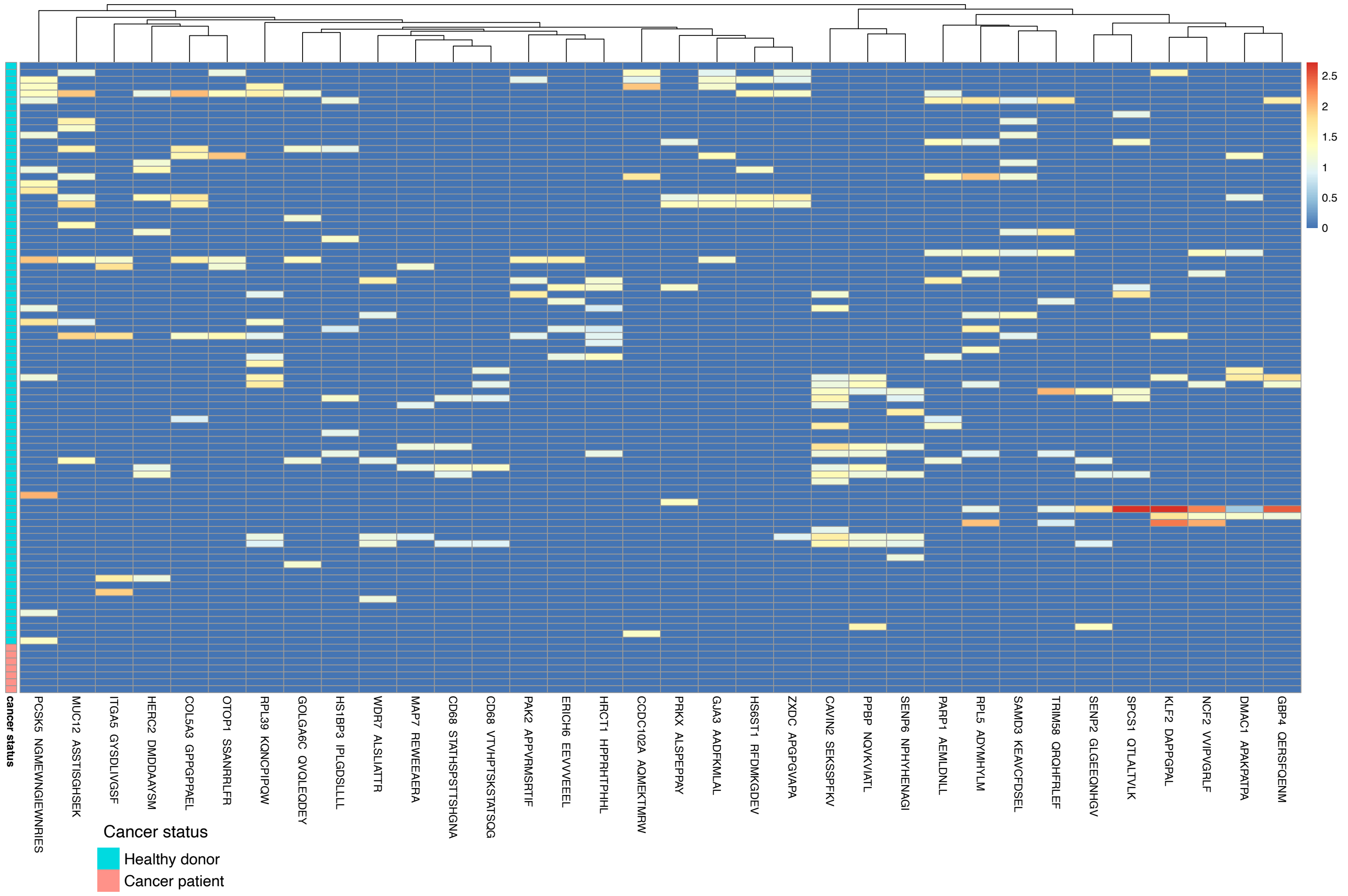

Figure S5

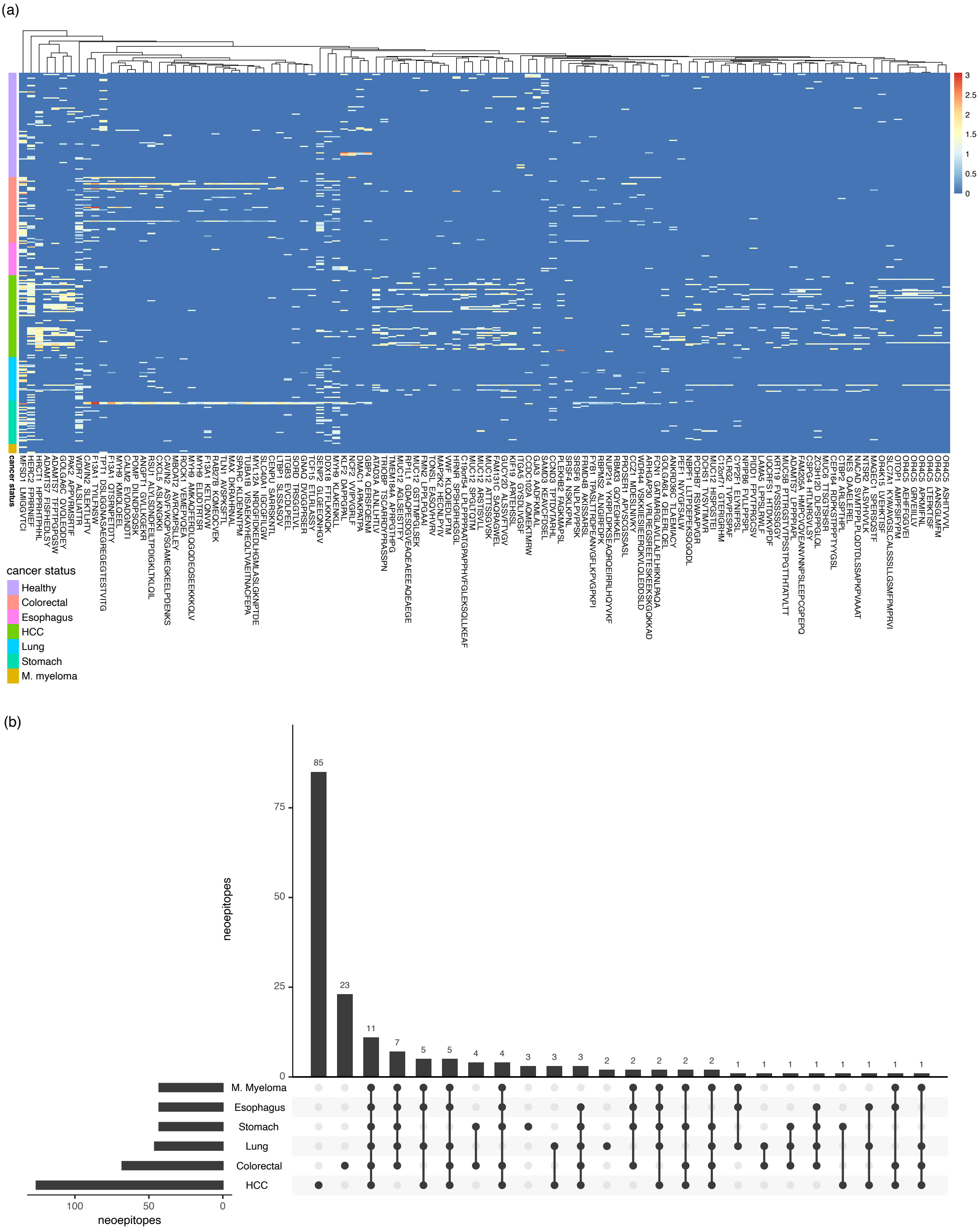
(a)
cancer status
Healthy
Colorectal
Esophagus
HCC
Lung
Stomach
M. myeloma
3
2.5
2
1.5
1
0.5
0
(b)
neoepitopes
85
23
11
7
5
5
4
4
3
3
3
2
2
2
2
2
1
1
1
1
1
1
1
1
0
25
50
75
M. Myeloma
Esophagus
Stomach
Lung
Colorectal
HCC
100
50
0
neoepitopes

Figure S6

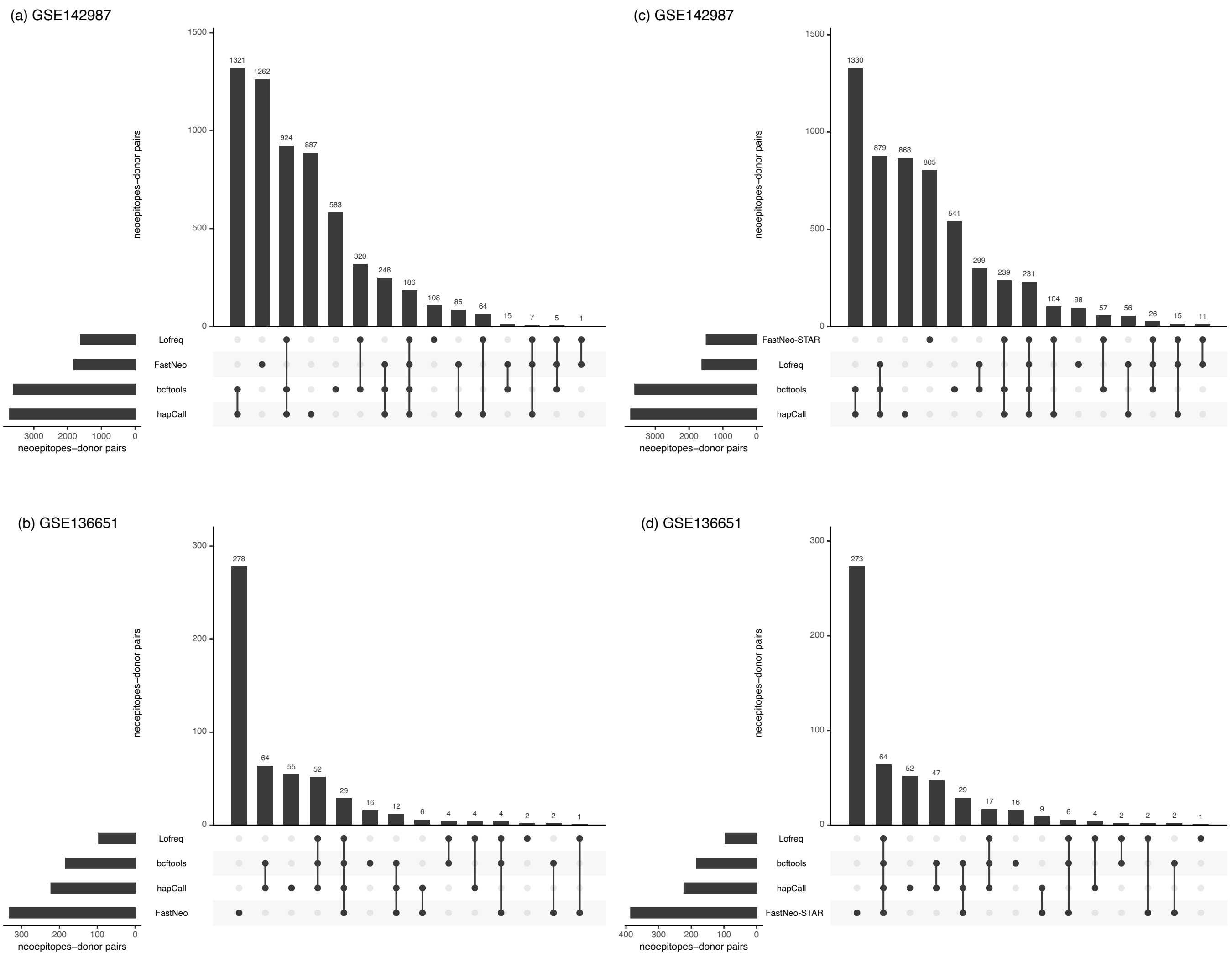

Figure S7

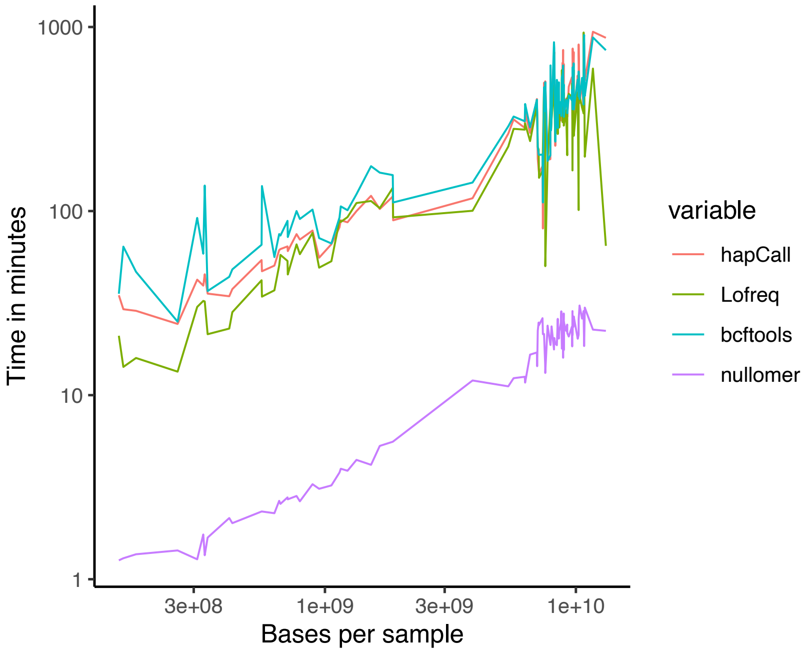

Figure S8

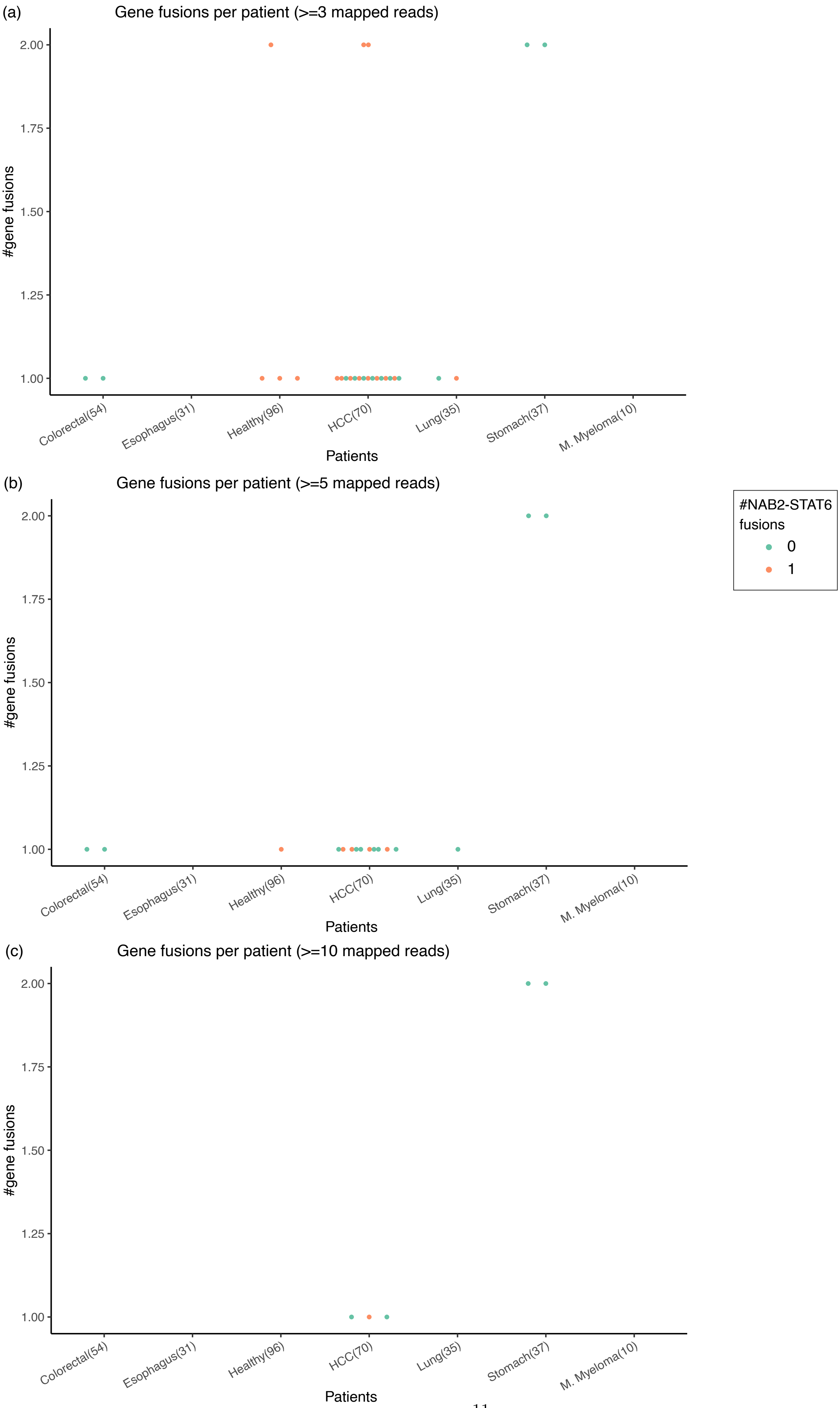
(a)
Gene fusions per patient (>=3 mapped reads)
(b)
Gene fusions per patient (>=5 mapped reads)
(c)
Gene fusions per patient (>=10 mapped reads)
#gene fusions
2.00
1.75
1.50
1.25
1.00
Colorectal(54)
Esophagus(31)
Healthy(96)
HCC(70)
Lung(35)
Stomach(37)
M. Myeloma(10)
Patients
#NAB2-STAT6 fusions
0
1

Figure S9

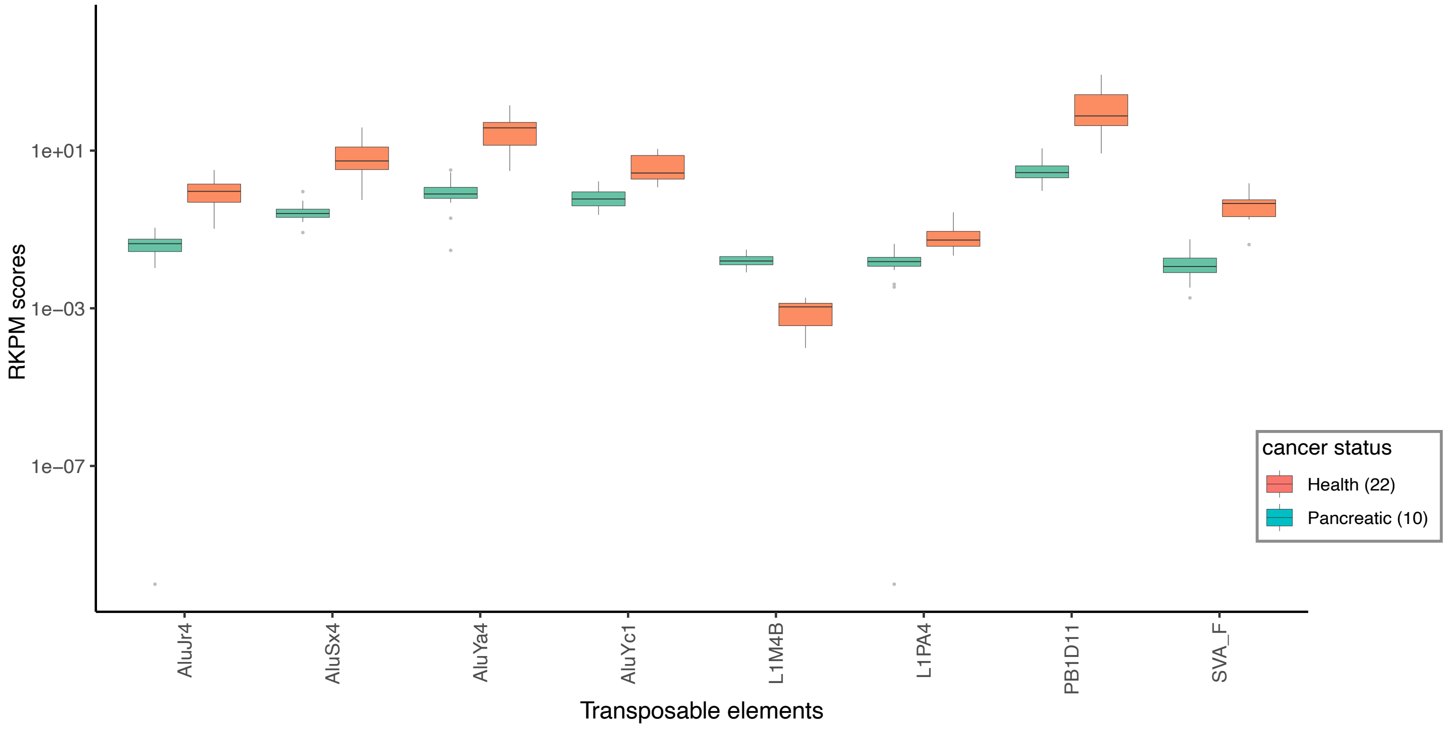

Figure S10

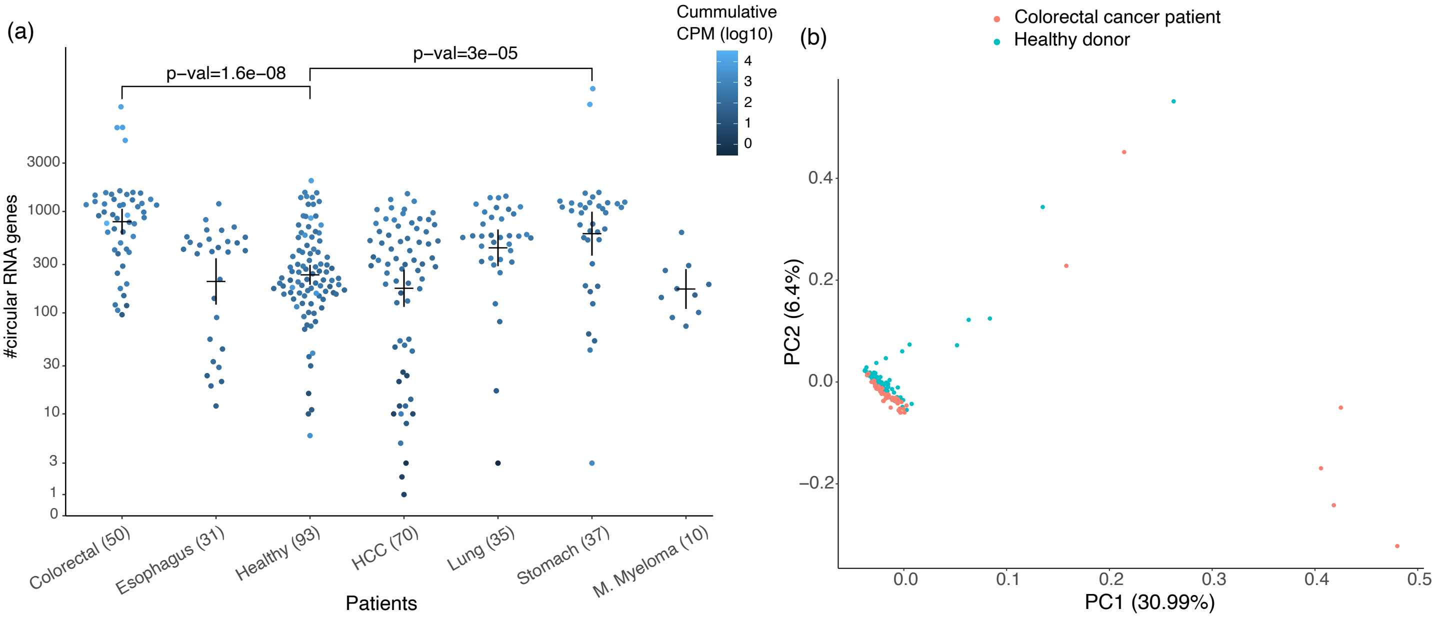

Figure S11

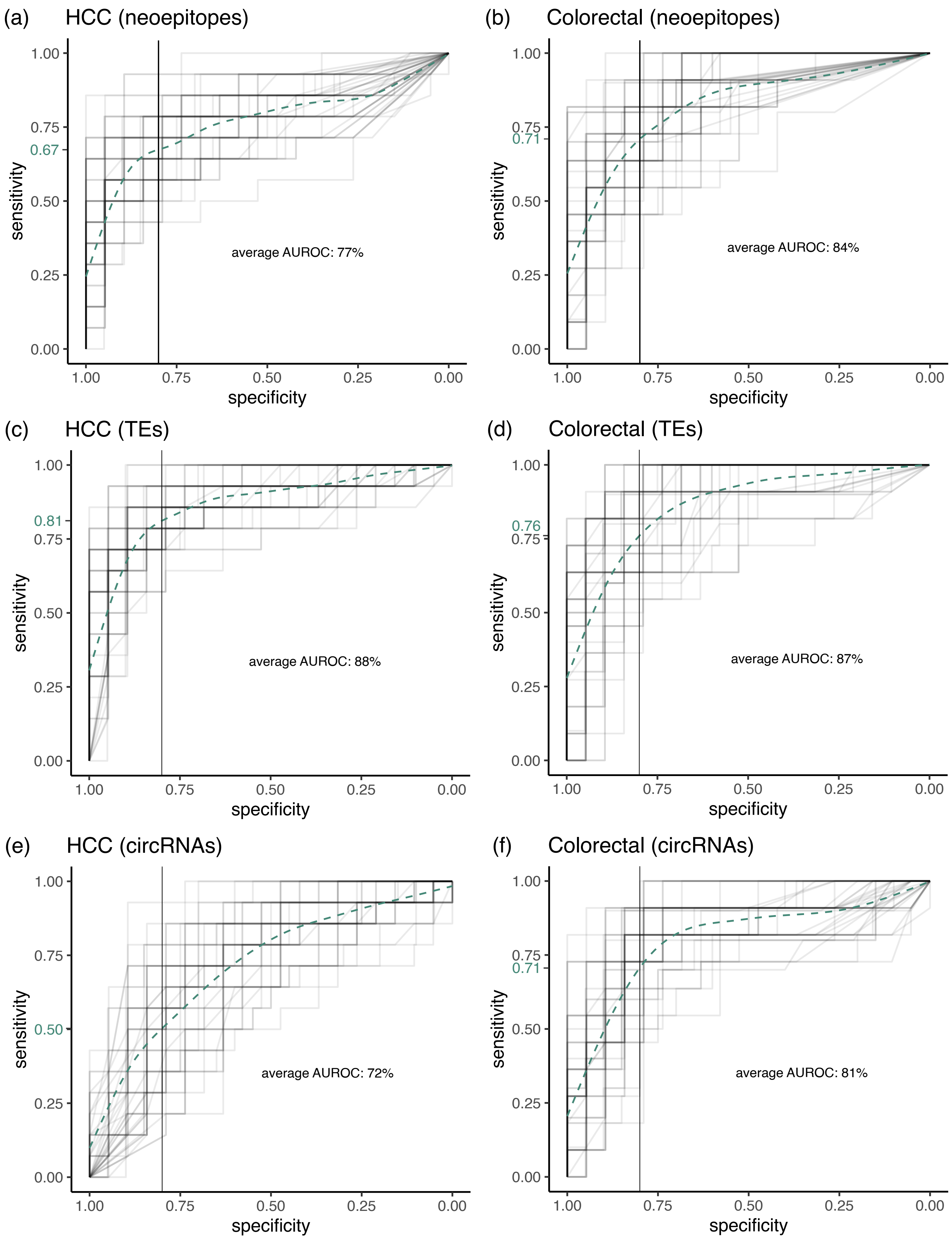
(a)
HCC (neoepitopes)
1.00
0.75
0.67
0.50
0.25
0.00
sensitivity
average AUROC: 77%
1.00
0.75
0.50
0.25
0.00
specificity
(b)
Colorectal (neoepitopes)
1.00
0.75
0.71
0.50
0.25
0.00
sensitivity
average AUROC: 84%
1.00
0.75
0.50
0.25
0.00
specificity
(c)
HCC (TEs)
1.00
0.81
0.75
0.50
0.25
0.00
sensitivity
average AUROC: 88%
1.00
0.75
0.50
0.25
0.00
specificity
(d)
Colorectal (TEs)
1.00
0.76
0.75
0.50
0.25
0.00
sensitivity
average AUROC: 87%
1.00
0.75
0.50
0.25
0.00
specificity
(e)
HCC (circRNAs)
1.00
0.75
0.50
0.25
0.00
sensitivity
average AUROC: 72%
1.00
0.75
0.50
0.25
0.00
specificity
(f)
Colorectal (circRNAs)
1.00
0.75
0.71
0.50
0.25
0.00
sensitivity
average AUROC: 81%
1.00
0.75
0.50
0.25
0.00
specificity

Figure S11

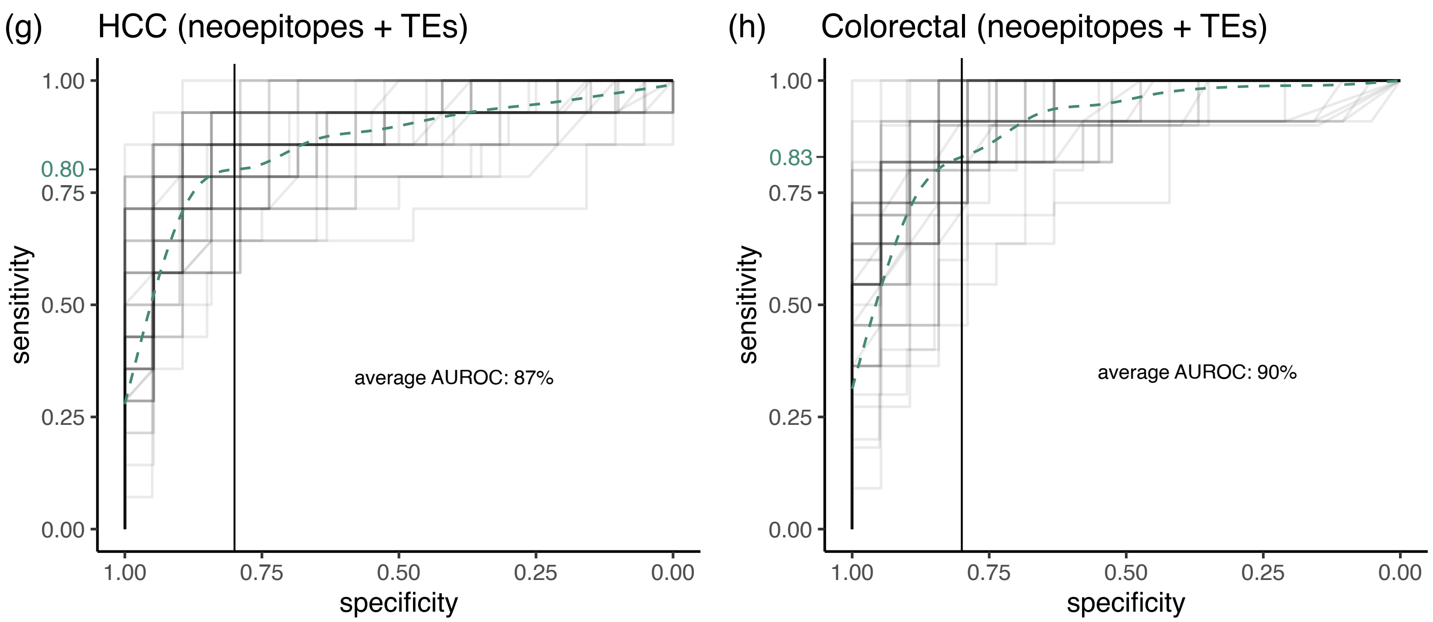
(g)
HCC (neoepitopes + TEs)
1.00
0.80
0.75
0.50
0.25
0.00
sensitivity
average AUROC: 87%
1.00
0.75
0.50
0.25
0.00
specificity
(h)
Colorectal (neoepitopes + TEs)
1.00
0.83
0.75
0.50
0.25
0.00
sensitivity
average AUROC: 90%
1.00
0.75
0.50
0.25
0.00
specificity
